# Voluntary vs. compulsory student evaluation of clerkships: effect on validity and potential bias

**DOI:** 10.1186/s12909-017-1116-8

**Published:** 2018-01-05

**Authors:** Sola Aoun Bahous, Pascale Salameh, Angelique Salloum, Wael Salameh, Yoon Soo Park, Ara Tekian

**Affiliations:** 1 Lebanese American University School of Medicine, Byblos, Lebanon; 2 Lebanese American University School of Pharmacy, Byblos, Lebanon; 3 Department of Medical Education, College of Medicine, University of Illinois at Chicago, Chicago, IL USA; 4 Lebanese American University Medical Center – Rizk Hospital, May Zahhar Street, Ashrafieh, P.O. Box: 11-3288, Beirut, Lebanon

**Keywords:** Clerkship, Evaluation, Rating, Authority, Bias

## Abstract

**Background:**

Students evaluations of their learning experiences can provide a useful source of information about clerkship effectiveness in undergraduate medical education. However, low response rates in clerkship evaluation surveys remain an important limitation. This study examined the impact of increasing response rates using a compulsory approach on validity evidence.

**Methods:**

Data included 192 responses obtained *voluntarily* from 49 third-year students in 2014–2015, and 171 responses obtained *compulsorily* from 49 students in the first six months of the consecutive year at one medical school in Lebanon. Evidence supporting internal structure and response process validity was compared between the two administration modalities. The authors also tested for potential bias introduced by the use of the compulsory approach by examining students’ responses to a sham item that was added to the last survey administration.

**Results:**

Response rates increased from 56% in the voluntary group to 100% in the compulsory group (*P* < 0.001). Students in both groups provided comparable clerkship rating except for one clerkship that received higher rating in the voluntary group (*P* = 0.02). Respondents in the voluntary group had higher academic performance compared to the compulsory group but this difference diminished when whole class grades were compared. Reliability of ratings was adequately high and comparable between the two consecutive years. Testing for non-response bias in the voluntary group showed that females were more frequent responders in two clerkships. Testing for authority-induced bias revealed that students might complete the evaluation randomly without attention to content.

**Conclusions:**

While increasing response rates is often a policy requirement aimed to improve the credibility of ratings, using authority to enforce responses may not increase reliability and can raise concerns over the meaningfulness of the evaluation. Administrators are urged to consider not only response rates, but also representativeness and quality of responses in administering evaluation surveys.

**Electronic supplementary material:**

The online version of this article (10.1186/s12909-017-1116-8) contains supplementary material, which is available to authorized users.

## Background

Student evaluation of instruction is widely embraced by educational programs as a measure of teaching and program effectiveness [1–3]. Ratings completed by students can often influence decisions about faculty promotion and tenure, and prompt curricular changes, highlighting the necessity of capturing accurate and meaningful students evaluations [4, 5]. Overall, most studies examining construct-related validity and consequential validity yielded positive results supporting the use of student ratings [6–9]. However, a few qualitative studies indicated that ratings are influenced more by student satisfaction than by objective measures of teaching quality [10–12]. Therefore, research studies have explored and identified features associated with increased utility and effectiveness of student ratings. These features are related to the response rate, structure of the evaluation instrument, its administration modality, and to the analysis of generated data [13, 14]. Reliability studies have demonstrated that averages of students’ ratings are more reliable than individual ratings, and that reliability is related to class size and response rate. Classes with less than 10 students or a response rate lower than 66% introduce a sampling bias and are associated with low reliability of ratings [1, 15, 16]. More recently, Phillips et al. [17] suggested that non-respondents characteristics, in addition to their number, should be examined for potential bias in any type of survey before results are interpreted.

In medical education, students’ perceptions of their learning experiences are instrumental for program improvement. Engaging students to provide thoughtful and attentive evaluations has been a real challenge, especially considering the burden that the increasing number of evaluations imposes on students during their medical studies [14]. Low response rates has been frequently reported as the main reason limiting meaningful interpretation of teaching evaluations because they can introduce sampling bias [18, 19]. On the other hand, stimulating responses may be associated with a quality bias and threat to validity.

Measures to improve response rates were considerably examined in the literature and they include adjusting delivery modalities to context [20–22], sending reminders, providing incentives [23, 24], ensuring student confidentiality, communicating expectations, personalizing request [25] and using authority [26]. Although these interventions were associated with improved response rates, the quality of rating was questioned only in incentive-induced responses [23, 27, 28] with more favorable ratings observed in incentive-based surveys [28, 29]. Thus, it is unclear whether increasing responses, thereby improving reliability, can directly translate into meaningful ratings that can enhance the validity of evaluations, particularly when authority is used to enforce responses. Therefore, the aims of this study were twofold: (1) to examine validity evidence related to response process and internal structure of our clerkship evaluation, and (2) to investigate the effect of using authority on response process, internal structure, and consequential validity. We hypothesize that increasing responses using a compulsory approach to evaluation would introduce a quality bias when enforced students (who would elect not to participate) provide inattentive responses.

## Methods

### The educational program

The MD program at the Lebanese American University (LAU) follows the American model of medical education and matriculates between 45 and 55 students each year. The clinical years (Medicine years III and IV) offer a traditional clerkship model of clinical rotations. The third year consists of seven core clinical clerkships including internal medicine, surgery, pediatrics, obstetrics and gynecology (Ob-Gyn), primary care, neurology, and psychiatry. These clerkships are distributed throughout the academic year and students rotate on different clerkships in pre-established groups. The fourth clinical year consists of more specialized clerkships, selective rotations, and electives. Students anonymously complete an evaluation of the clerkship and teachers within the first two weeks following completion of each rotation. Participation in this evaluation had been voluntary since the inception of the school (in 2009) until the start of the 2015–2016 academic year when participation has become compulsory, linked to grade release, aiming at increasing response rate. Only clerkship evaluation (and not teacher evaluation) was used in this study.

### The clerkship evaluation instrument

A committee of seven educational experts developed the clerkship evaluation form that was used for all clerkships. Over the years, changes suggested by faculty and students have been made to the evaluation process and have affected the instrument itself and administration modality. These changes included shortening of the instrument and adoption of an online modality with frequent reminders. The final instrument used since 2014 (without any further change) consists of thirteen items that capture students’ rating on a five-point Likert scale (Strongly Agree = 5 to Strongly Disagree = 1). These items measure three aspects of the clerkship: organization and structure, teaching activities, and learning environment. Neither the evaluation instrument, nor the administration modality have changed since 2014, except for the compulsory aspect applied in 2015. (The complete form is available as Additional file 1).

### Database

We used evaluation responses of third year medical students provided during the academic year 2014–2015 (class of 2016) and the first half of the academic year 2015–2016 (class of 2017) to gather validity evidence relating to response process, internal structure and consequences of compulsory approach. Each class consisted of 49 students rotating in small groups. Evaluations during the academic year 2014–2015 were anonymous, voluntary and administered online at the end of each clerkship. The evaluation software allows the identification of response status of students (respondents vs. non-respondents) in each clerkship, without any information about individual ratings (evaluations did not include any student identifier, and the generated report includes aggregated information about the clerkship). This facilitated the analysis of non-response bias (defined below) in the voluntary group and of consistency of responses across clerkships. Evaluation information is managed at the Dean’s Office, and all data used in this study, whether individual (response status) or aggregate (scores, ratings, etc.) was de-identified by the Office administrator before it is made available to investigators. Collected information included student characteristics (age and gender), in addition to their aggregate grades by clerkship. Given the anonymous nature of the evaluation, individual students’ grades could not be obtained, and hence could not be linked with their evaluation ratings. Student responses provided during the first six months of the academic year 2015–2016 (class of 2017; when participation became compulsory) were used to conduct comparisons with students from the previous academic year. The Lebanese American University Institutional Review Board (IRB) approved the exempt status of this study because it involves the analysis of existing data in a manner that subjects cannot be identified in anyway. Furthermore, the LAU IRB judged that consent is unnecessary given the nature of the study. A representation of the study design is available as Additional file 2.

### Validity conceptual framework

The validity framework described by Messick [30] embraces a unitary approach from five sources of validity evidence: *content*, *response process*, *internal structure*, *relations to other variables*, and *consequences of testing*. In this study, we examined response process, internal structure, and consequential validity evidence in relation to voluntary and compulsory participation in clerkship evaluation. We tested response process validity evidence using two factors: response rate and non-response bias. *Response Rate* (RR) was estimated using the six American Association of Public Opinion Research (AAPOR) (2011) definitions [31]. We adopted AAPOR RR6 definition because all non-respondents were eligible for participation (RR6 = (complete surveys + incomplete surveys)/all eligible subjects). *Non-response bias* corresponds to the bias introduced by non-respondents if one of more of their characteristics could have affected observed results. For example, when all females in a class do not complete clerkship evaluation, the responses obtained (all from males) do not represent an adequate class sample; and responses from females might (or might not) yield different results [17]. We estimated non-response bias in our study by comparing characteristics between respondents and non-respondents (age, gender, and grades) in the voluntary group. This bias does not apply to the compulsory group.

Internal structure was assessed using exploratory factor analysis to identify a latent structure supporting student responses. Further, Cronbach’s alpha was used to estimate the internal-consistency reliability of obtained evaluation rating data. We defined consequential validity evidence as the change in the evaluation scores and the bias introduced by the application of the compulsory approach using authority (authority-induced bias). In this study we hypothesized that the compulsory nature of the evaluation would introduce a quality bias despite the increased number of obtained responses; students who are not voluntarily interested in evaluating clerkship effectiveness may provide oblivious, unrepresentative rating. Concerning the directionality of the expected score change, there is not enough research data to suggest that compulsory ratings would be more or less positive compared to those obtained from voluntary participation. Since the change to the administration modality (compulsory) was implemented at the beginning of the 2015–2016 academic year, no comparisons between modalities in the same cohort of students was possible. To test the quality bias hypothesis, we intended to examine students’ attention to each item, with the consideration that students who complete the evaluation compulsorily, would not be attentive to item content and may complete the form randomly. For that purpose, we added to the form of the last invitation prior to this study (December 2015) an irrelevant item, unrelated to clerkship effectiveness: “The clerkship helped me in my application to the bank”. This item was included in the middle of the form among other items and had the same scale options. This intervention was administered to a single cohort during the 2015–2016 (compulsory modality) academic year. This positioning was selected to occur after students were familiar with the compulsory process. We felt it was not appropriate to continue with this intervention as students may start to notice the sham question and alter their responses to the entire survey. Answers to the sham item were examined and positive ratings (Agree and Strongly Agree) were considered representative of a potential bias because they implicitly indicate that the student was not attentive to the content. This assumption was confirmed in a formal feedback session with the whole class where each student described his/her answer to this item anonymously using a paper-based survey (The complete survey is available as Additional file 3).

### Statistical analysis

Statistical analysis was conducted using SPSS version 21.0 for Windows (SPSS Inc., Chicago, USA). We used descriptive statistics to determine response rates, student characteristics, student ratings, and group and class averages of students’ clerkship grades. Data were summarized as frequencies and percentages for categorical variables and means (SD) for ordinal and continuous variables (clerkship rating being the sum of item grades with a maximum of 65 points). Two-tailed unpaired *t* tests were used to compare means of ratings and grades between the voluntary and compulsory groups, and ANOVA for comparison of means across clerkships and groups. However, since responses in different clerkships within the same cohort are dependent, instead of being independent (because common students rate the different clerkships), a more direct analysis using for example mixed-effects regression should have been used. This statistical analysis necessitates the identification of individual responses per student as repeated measures, which is impossible in our study design, where participation in clerkship evaluations is anonymous. Therefore, we analyzed our data as non-repeated, knowing that this reduces the power to detect a difference but does not normally lead to type I error as long as the analysis does not involve the larger sample obtained from repeated responses. Chi-squared tests were used to compare categorical data. Cronbach’s alpha was used to determine consistency of response/non-response per individual across clerkships in the voluntary group, and as a reliability estimate. Spearman’s coefficient was used as a measure of correlation between students’ ratings of clerkships and their grades. We conducted an exploratory factor analysis on responses from both voluntary and compulsory evaluations to determine the structure underlying students’ responses, using varimax rotation. Kaiser-Meyer-Olkin was used to determine sample adequacy. Factor loadings greater than 0.4 was considered significant for retention.

## Results

1. Response process validity evidence:
  - Response rates and characteristics of responses in the voluntary group (Table 1):
    The class of 2016 consisted of 49 students. Invitations to participate were sent to all eligible students according to their clerkships; 343 invitations were sent throughout the year and 192 responses were collected, yielding an overall response rate (AAPOR RR 6) of 56%. Response rates (AAPOR RR 6) varied between 45% and 63.3% across clerkships with no statistically significant differences. The consistency of respondent/non-respondent status was 0.84. Average clerkship ratings were significantly different between clerkships (*P* = 0.01), with highest ratings given for the Ob-Gyn clerkship and lowest for Surgery. The presence of comments was comparable across clerkships. Aggregate students’ grades (another measure of clerkship effectiveness) were comparable across clerkships and there was no significant correlation between clerkship rating (provided by the student) and aggregate students’ grades.
  - Comparison of clerkship evaluation between the voluntary and compulsory groups (Table 2):
    The class of 2017 consisted of 49 students. Data were collected for the first six months of the consecutive academic year and 171 invitations were sent, with a RR of 100%. Response rates were significantly different between the two consecutive years (*P* < 0.001). However, ratings were similar for most clerkships except the Ob-Gyn where compulsory evaluation was associated with lower average clerkship rating (50.3 vs. 56.6, *P* = 0.02). On the other hand, aggregate students’ grades were significantly higher in the voluntary group (*P* = 0.001), while the presence of comments was comparable between the two groups.
  - Non-response bias (Table 3):
    Table 3 summarizes characteristics of students who provided clerkship evaluation (respondents) and those who did not (non-respondents) in the voluntary group. Both sub-groups had comparable ages and grades. However, although females represented 41% of the whole class, they had significantly greater participation only in primary care (53% vs. 21%, *P* < 0.05) and psychiatry (54% vs. 24%, *P* < 0.05).
2. Internal structure validity evidence: (Table 4):
  Exploratory factor analysis was conducted on all clerkship evaluation responses. The analysis yielded two factors explaining 66.19% of the total variance for the entire dataset. Factor 1 was labeled learning environment and activities and it explained 57.01% of the total variance, while factor 2 was labeled adequacy of the site for the clerkship and it explained 9.18% of the total variance. The reliability of the evaluation form was 0.935.
3. Consequential validity evidence: authority-induced bias (Table 5):
  Authority-induced bias was tested only in the final cohort where the sham question was added to the clerkship evaluation form. As previously stated, answers with positive rating (Strongly Agree and Agree) were considered biased because they implicitly indicate that the student was not attentive to the content. Fourteen students (32.56%) provided positive ratings and were considered biased, while 29 responses (67.44%) were considered unbiased (six students were on vacation). Average ratings were comparable between the voluntary group, unbiased compulsory group, and biased compulsory group. However, ratings were consistently lower in the biased group though this difference did not reach statistical significance. Reliability of ratings was comparable across groups.

**Table 1:** Response rates and characteristics of responses in various clerkships in the voluntary group

	IM *N* = 49	Surgery N = 49	Pediatrics N = 49	Ob-Gyn N = 49	PC N = 49	Neurology N = 49	Psychiatry N = 49	All *N* = 343	*P*
Response Rate [N (%)]	31(63.3%)	28(57.1%)	23(46.9%)	22(44.9%)	30(61.2%)	30(61.2%)	28(57.1%)	192(56.0%)	NS
Average Rating^a^ Mean (SD)	53.77(7.92)	49.70(7.93)	55.83(7.90)	56.60(6.59)	55.33(8.88)	49.86(9.92)	52.33(8.92)	53.19(8.69)	0.01
Presence of Comments [N (%)]	8(25.8%)	8(28.6%)	6(26.1%)	2(9.1%)	4(13.3%)	10(33.3%)	6(21.4%)	44(22.9%)	0.36
Aggregate grades of respondents Mean (SD)	77.51(4.49)	79.50(2.93)	79.26(4.16)	80.11(3.79)	77.96(4.88)	78.26(4.32)	84.60(4.03)	79.53(4.09)	0.25

*Abbreviations: IM* Internal Medicine, *Ob-Gyn* Obstetrics and Gynecology, *PC* Primary Care

^a^Clerkship rating corresponded to the sum of items ratings per student per clerkship (maximum rating that can be obtained is 65). All values were averaged

**Table 2:** Comparison of clerkship evaluations between voluntary (class of 2016) and compulsory (class of 2017) approaches

Characteristics	Voluntary (*N* = 192 responses)	Compulsory (*N* = 171 responses)	P
Response rate	192/343 (56.0%)	171/171 (100%)	< 0.001
Overall Rating^a^ [Mean (SD)]			
Internal Medicine	53.77(7.91)	55.94(8.78)	0.38
Surgery	49.70(7.93)	50.33(4.50)	0.78
Pediatrics	55.83(7.90)	53.38(3.96)	0.21
Ob-Gyn	56.60(6.59)	50.32(9.67)	0.02
PC	55.33(8.88)	54.95(5.92)	0.86
Neurology	49.86(9.92)	51.43(8.48)	0.55
Psychiatry	52.33(8.92)	53.58(8.47)	0.65
All Clerkships	53.19(8.69)	52.90(7.63)	0.76
Presence of Comments^b^ [N (%)]	44(22.9%)	42(24.6%)	0.71
Aggregate grades of respondents [Mean (SD)]	79.53 (4.09)	78.76 (3.51)	0.001

*Abbreviation: Ob-Gyn* Obstetrics and Gynecology

^a^Overall rating per clerkship was calculated for respondents, where the number of obtained evaluations per clerkship is less than 49 (the total number of students in the class) in the voluntary group, depending on the response rate per clerkship, and equal to 49 in the compulsory group

^b^Comments were examined on the whole number of obtained responses in each group

**Table 3:** Characteristics of respondents vs. non-respondents in the voluntary group (class of 2016): Non-response bias

Characteristics	Internal Medicine		Surgery		Pediatrics		Ob-Gyn		Primary Care		Neurology		Psychiatry	
	*R* *N = 31*	*NR* *N = 18*	*R* *N = 28*	*NR* *N = 21*	*R* *N = 23*	*NR* *N = 26*	*R* *N = 22*	*NR* *N = 27*	*R* *N = 30*	*NR* *N = 19*	*R* *N = 30*	*NR* *N = 19*	*R* *N = 28*	*NR* *N = 21*
Female Gender n (%)	15(48%)	5(28%)	13(46%)	7(33%)	10(43%)	10(38%)	12(55%)	8(30%)	16(53%)	4(21%)^a^	13(43%)	7(37%)	15(54%)	5(24%)^a^
Age [Mean(SD)]	25.1(1.0)	25.1(0.9)	25.2(1.1)	24.9(0.8)	25.1(1.1)	25.1(0.9)	25.0(1.1)	25.2(0.9)	24.9(1.0)	25.3(1.0)	25.2(1.1)	24.9(0.8)	25.0(1.0)	25.2(1.0)
Aggregate students’ grades [Mean(SD)]	77.51 (4.49)	79.34 (3.63)	79.50 (2.93)	78.98 (2.20)	79.26 (4.16)	79.91 (3.34)	80.11 (3.79)	80.78 (3.65)	77.96 (4.88)	79.48 (2.85)	78.26 (4.32)	79.43 (3.45)	84.60 (4.03)	83.14 (2.95)

*Abbreviations: R* respondents, *NR* non-respondents, *Ob-Gyn* Obstetrics and Gynecology

^a^*P* < 0.05;

**Table 4:** Exploratory Factor Analysis: Item-level factor loading^a^

Question number and item	Factor 1	Factor 2	Total scale correlation^b^
Q3 The clerkship facilitated development of knowledge and skills necessary to take an accurate history, perform a thorough physical examination and formulate an appropriate differential diagnosis	**0.804**	0.089	0.701
Q4 The clerkship introduced the student to basic principles of management	**0.741**	0.269	0.755
Q2 The clerkship introduced the student to clinical diseases	**0.739**	0.309	0.769
Q9 The teaching activities (rounds, lectures, tutorials, bed-side teaching, case discussions…) improved my overall knowledge	**0.738**	0.251	0.744
Q6 The assessment process reflected the learning objectives	**0.729**	0.359	0.803
Q10 The learning environment was safe and conducive to learning	**0.721**	0.322	0.769
Q11 Feedback was given in an effective manner during this rotation	**0.718**	0.254	0.742
Q1 Clear learning objectives for this clerkship were provided	**0.712**	0.382	0.798
Q5 The clerkship encouraged the student to take an active role as a member of the healthcare team, acquire professional attitudes, and develop competencies in communication skills and coordinated care	**0.675**	0.341	0.745
Q12 The clerkship was overall well organized	**0.629**	0.466	0.785
Q7 The clerkship provided enough opportunities to meet the number of required clinical encounters	0.217	**0.868**	0.686
Q13 The clinical site is appropriate for this clerkship	0.330	**0.827**	0.754
Q8 The clerkship provided enough opportunities to learn and practice clinical skills	0.322	**0.826**	0.747
Descriptive results			
Mean (SD) Year 2014–2015	41.41(6.64)	11.81(2.51)	53.19(8.69)
Mean (SD) Year 2015–2016	41.11(5.95)	11.94(2.45)	52.90(7.63)
Mean (SD) Total	41.29(6.36)	11.87(2.48)	53.07(8.26)
Reliability results			
Cronbach’s Alpha 2014–2015	0.934	0.873	0.945
Cronbach’s Alpha 2015–2016	0.909	0.885	0.916
Cronbach’s Alpha Total	0.925	0.876	0.935

^a^Bold numbers represent significant item loading on factors

^b^*P* values for all correlation coefficients <0.001

**Table 5:** Effect of authority-induced bias on response pattern of students in the compulsory group^a^

Characteristics	Voluntary (*N* = 192 responses)	Compulsory – Non Biased (*N* = 29 responses)	Compulsory – Biased (*N* = 14 responses)	P
Overall rating^b^ [Mean (SD)]				
Internal Medicine	53.77(7.92)	59.21(6.00)	58.96(5.85)	0.23
Surgery	49.70(7.93)	54.04(4.91)	40.71(4.95)	0.10
Pediatrics	55.83(7.90)	59.21(4.36)	49.20(7.83)	0.36
Ob-Gyn	56.60(6.59)	55.60(6.26)	49.83(15.15)	0.32
Primary Care	55.33(8.88)	57.87(5.51)	51.21(−--)^c^	0.79
Neurology	49.86(9.92)	52.46(9.95)	46.20(−--)^c^	0.82
Psychiatry	52.33(8.92)	58.71(6.40)	–	0.19
All Clerkships	53.19(8.69)	56.38(6.17)	50.88(10.09)	0.09
Presence of Comments^d^ [N (%)]	44(22.9%)	5(17.2%)	1(7.1%)	0.32
Cronbach alpha	0.945	0.946	0.944	NA

*Abbreviations: Ob-Gyn* Obstetrics and Gynecology, *NA* not applicable

^a^Comparisons involved only the last evaluation administration (including the sham question) in the compulsory group

^b^Overall rating per clerkship was calculated for respondents in the voluntary group, where the number of obtained evaluations per clerkship is less than 49 (the total number of students in the class) depending on the response rate per clerkship

^c^No standard deviation is computed because only one student completed the evaluation for Primary Care

^d^Comments in the voluntary group were examined on the whole number of obtained responses (192)

## Discussion

The major findings from this study are as follows: 1) the reliability of ratings was adequate despite the low response rate, and 2) improving response rate using the compulsory approach did not improve reliability, and was associated with inattentive responses in 32.6% of cases without yielding different ratings.

Students’ evaluation of their learning experiences is normally used for several purposes. Administrators use them to make decisions (faculty promotion, incentives), faculty consider them to improve their teaching, and institutions include them as indicators of program effectiveness [5, 8, 9]. Given the high-stake use of these ratings, interpretation should be made carefully, considering quality psychometric measures. Validity evidence has been examined in the literature using correlation studies linking student ratings to other measures of effective teaching (e.g. academic achievement of students) [32, 33]. However, the practical utility of evaluations largely depends on adequate samples (response rates) [1, 15, 16]. Therefore, increasing response rates has confronted researchers for decades. More recently, Phillips et al. [17] described the concept of non-response bias, defined as a bias, or threat to validity, introduced by non-respondents if one or more of their characteristics would have affected rating shall they respond to the survey. A class sample is representative not only when its size exceeds two thirds of the class, but additionally when it captures most of the diversities in that class. Therefore, increasing responses is aimed at adding up information that is meaningful for the purpose of its use. A systematic review by VanGeest et al. [24] on strategies to improve response rates in physicians surveys suggested that both incentive and design-based approaches are effective in stimulating more responses, and that non-response bias can be ignored in homogeneous populations. However, artificially improving response rates may lead to inattentive responses and hence present a qualitative threat to the utility of evaluations. Interestingly, the association between response rates and the provided rating scores remains largely unaddressed in the literature despite few small studies showing higher scores obtained in incentive-based surveys [28, 29].

Authority-based surveys were not evaluated in the literature for the possibility of bias introduced by respondents, who would have otherwise been non-respondents. Our study showed that although response rates were below suggested standards in voluntary surveys, the obtained ratings were reliable. Furthermore, there was a consistency of participation per individual across clerkships, whereby responses were largely provided by the same students in each clerkship. Moreover, enforcing responses using authority expectedly yielded higher response rate but did not improve the reliability of rating. Furthermore, while improving response rates is normally associated with increasing number of comments that may have meaning and be actionable, this was not the case in our study where the number of comments did not increase significantly in the compulsory group. Interestingly, the ratings were comparable between the voluntary and compulsory groups except for one clerkship, and therefore, affecting the willingness of students to participate in clerkship evaluation using authority did not yield different ratings. One possible confounder in this comparison was students’ academic performance. Students in the voluntary group had significantly higher aggregate grades than in the compulsory group. To adjust for this confounder, we compared grade averages of the whole class between the two cohorts and found no statistically significant difference.

To explore the risk of quality bias introduced by enforced participations, we examined students’ responses to the sham question and were able to identify 14 (32.56%) biased participations. Comparing these evaluations to others provided by non-biased participations and those from the voluntary group, we did not find any statistically significant difference (despite a non-significant tendency toward lower ratings of clerkships in the biased group, and toward a lower availability of comments). However, two drawbacks exist in this approach. First, the number of students in the compulsory group could be higher, thereby increasing the power of the study to detect meaningful differences and allow accurate conclusions. Second, although it may be relatively easy to assess non-response bias, it is clearly very challenging to predict which students would refuse to participate should the participation be voluntary. However, this is the first study to our knowledge that examined the effect of a compulsory approach on clerkship evaluation and to show that around a third of responders acknowledged that they provided random, inattentive ratings. Interestingly, These ratings were not significantly different from unbiased and from voluntary participations. On the other hand, students who noticed the sham question found it very unreasonable and considered this to be a technical problem in the evaluation system.

Other aspects of clerkship evaluation to be considered include context specificity of learning, academic performance of students, and characteristics of non-respondents in voluntary participations. Our study showed that students rated differently their learning experiences across clerkships, which is consistent with the literature [34]. Interestingly, their aggregate grades were comparable across clerkships, although it is generally suggested that high ratings are associated with high performance [7]. However, since our evaluation is anonymous, individual grades were not available and conclusions in that regard cannot be drawn with accuracy. When non-respondents’ characteristics were examined to identify bias, there were more female respondents than non-respondents, and this was significant in two out of seven clerkships. Therefore, a possible gender bias is to be considered in the voluntary group only if females are expected to provide different ratings by comparison to males. While some research studies found that female gender was associated with more positive rating, our study was not conclusive in this regard because of the anonymous nature of the evaluation [10, 35, 36].

This study has many limitations. First, we compared voluntary and compulsory approaches to capture student responses using two different cohorts; hence conclusions should be carefully drawn in that regard. However, clerkship types, educational activities, teachers, class size, students’ average age, gender distribution, and academic achievement were overall comparable, based on bias analysis results comparing characteristics between the two class cohorts. Furthermore, a comparison of academic achievement across classes in previous years (before the study) showed similar variation in overall scores. A second limitation is related to the small sample size and monocentric model (single institution experience) of the study, which may introduce a cultural bias and hence limit the generalizability of our results. However, the nature of the response rate problem being general, and the use of authority being common to many educational institutions make our study plausible for replication in different contexts. Another sampling limitation to be considered is the inclusion of a complete cohort in the first group (voluntary group) and half of a cohort in the second group (compulsory group). This purposeful approach, used to avoid possible effect of the intervention (sham item) on subsequent evaluations, could have introduced a sampling bias. Third, the fact that the response rate in the voluntary group was not considerably low and that the reliability of rating was high leads to a ceiling effect and makes a significant impact of increasing responses on reliability less likely. A fourth and major limitation is related to the statistical analysis involving clerkship comparisons within the same cohort. Conducted analyses, enforced by the study design, could not account for non-independency of provided ratings, hence reducing the power to detect a difference between clerkships. However, this aspect does not involve the key findings of our study. Finally, the small number of responses to the “bias” item could make conclusions about authority-induced bias unsure, and probably subject to change shall the sample size be larger. Further larger studies are needed to examine the effect of stimulating response using authority, on the quality of provided responses.

## Conclusions

In conclusion, our study concurred with literature findings that students’ ratings of their learning experiences yield reliable results. While response rates and characteristics of non-respondents should be examined before data interpretation is conducted, we propose that using authority to improve response rates may not always improve the reliability, does not yield different ratings, and could threaten the validity only if enforced evaluations were significantly different from voluntary evaluations. The latter needs to be confirmed in larger studies. Other methods that promote representative and attentive student responses should be explored especially if significant consequences are attached to these ratings.

## Additional files

Additional file 1: 2014/2015 Clerkship Evaluation Form. This file presents the clerkship evaluation form that was used in 2014–2015 by the Dean’s Office as a routine practice to collect students’ opinion about their learning experience during each clerkship. (DOCX 24 kb)

Additional file 2: Study Design. This file is a representation of the study conduct including information about cohort sizes, response rates, and administration of the bogus item (DOC 47 kb)

Additional file 3: December 2015 Clerkship Evaluation Follow-up Survey. This file presents the follow-up survey that was administered to students in the second cohort who completed the clerkship evaluation form with the bogus item (administered in December 2015). It intended to confirm the bias hypothesis (DOC 26 kb)

## Abbreviations

AAPOR: American Association of Public Opinion Research
LAU: Lebanese American University
Ob-Gyn: Obstetrics and gynecology
RR: Response rate
SD: Standard deviation
