# Supplementary material for: Voluntary vs. compulsory student evaluation of clerkships: effect on validity and potential bias
Source: BMC Med Educ. 2018 Jan 5;18:9. doi: 10.1186/s12909-017-1116-8 (PMC5756350; doi:10.1186/s12909-017-1116-8)
Supplement: Supplementary file 1 — 2014/2015 Clerkship Evaluation Form. This file presents the clerkship evaluation form that was used in 2014–2015 by the Dean’s Office as a routine practice to collect students’ opinion about their learning experience during each clerkship. (DOCX 24 kb) [file 12909_2017_1116_MOESM1_ESM.docx]

Dear Med III Students, thank you for taking some time to fill this clerkship evaluation. Please note that your comments are highly appreciated.

| **Marking Instructions:** ★ Mandatory Example: Correct Mark □ ☒ |
| --- |

| **EVALUATION OF THE CLERKSHIP** |
| --- |

| **Clerkship Name ……………………………………..** |
| --- |

| ★ | **1.** | Month(s) on clerkship: |
| --- | --- | --- |

| □ | June 30-August 24, 2014 |
| --- | --- |
| □ | August 25-October 19, 2014 |
| □ | October 20-December 14, 2014 |
| □ | December 15-February 8, 2015 |
| □ | February 9-April 5, 2015 |
| □ | April 6-May 31, 2015 |

| ★ | **2.** | Clerkship evaluation: |
| --- | --- | --- |
|  |  | Note: Please choose “Not Applicable” (N.A.) if a clear judgment cannot be made. |

|  |  | Strongly Agree | Agree | Neutral | Disagree | Strongly Disagree | N.A. |
| --- | --- | --- | --- | --- | --- | --- | --- |
| Clear learning objectives for this clerkship were provided. |  | □ | □ | □ | □ | □ | □ |
| The clerkship introduced the student to clinical diseases |  | □ | □ | □ | □ | □ | □ |
| The clerkship facilitated development of knowledge and skills necessary to take an accurate history, perform a thorough physical examination and formulate an appropriate differential diagnosis. |  | □ | □ | □ | □ | □ | □ |
| The clerkship introduced the student to basic principles of management. |  | □ | □ | □ | □ | □ | □ |
| The clerkship encouraged the student to take an active role as a member of the healthcare team, acquire professional attitudes, and develop competencies in communication skills and coordinated care. |  | □ | □ | □ | □ | □ | □ |
| The assessment process reflected the learning objectives. |  | □ | □ | □ | □ | □ | □ |
| The clerkship provided enough opportunities to meet the number of required clinical encounters. |  | □ | □ | □ | □ | □ | □ |
| The clerkship provided enough opportunities to learn and practice clinical skills. |  | □ | □ | □ | □ | □ | □ |
| The teaching activities (rounds, lectures, tutorials, bed-side teaching, case discussions…) improved my overall knowledge. |  | □ | □ | □ | □ | □ | □ |
| The learning environment was safe and conducive to learning. |  | □ | □ | □ | □ | □ | □ |
| Feedback was given in an effective manner during this rotation. |  | □ | □ | □ | □ | □ | □ |
| The clerkship was overall well organized. |  | □ | □ | □ | □ | □ | □ |
| The clinical site is appropriate for this clerkship. |  | □ | □ | □ | □ | □ | □ |

| ★ | **3.** | Which were useful as aids to learning in this rotation? |
| --- | --- | --- |
|  |  | Note: Check all that apply |

| □ | Wards |
| --- | --- |
| □ | Clinics |
| □ | Lectures |
| □ | Tutorials |
| □ | Attending Rounds |
| □ | Rounds with Residents |
| □ | Other Rounds (lab, radiology, test and ECG interpretation…) |
| □ | Morning Report |
| □ | Surgical acts |
| □ | Procedures |
| □ | Case Discussions |
| □ | Feedback from Residents |
| □ | Feedback from Attendings |
| □ | Mid-clerkship Feedback |
| □ | Mini-CEX |
| □ | Electronic Resources & Textbook |

|  | **5.** | Comments/suggestions for the Clerkship: |
| --- | --- | --- |

| █ █ █ █ █ █ █ █ █ █ █ █ █ █ █ █ █ █ █ █ █ █ █ █ █ █ █ █ █ █ █ █ █ █ █ |
| --- |
| █ █ █ █ █ █ █ █ █ █ █ █ █ █ █ █ █ █ █ █ █ █ █ █ █ █ █ █ █ █ █ █ █ █ █ |
| █ █ █ █ █ █ █ █ █ █ █ █ █ █ █ █ █ █ █ █ █ █ █ █ █ █ █ █ █ █ █ █ █ █ █ |
| █ █ █ █ █ █ █ █ █ █ █ █ █ █ █ █ █ █ █ █ █ █ █ █ █ █ █ █ █ █ █ █ █ █ █ |
| █ █ █ █ █ █ █ █ █ █ █ █ █ █ █ █ █ █ █ █ █ █ █ █ █ █ █ █ █ █ █ █ █ █ █ |
| █ █ █ █ █ █ █ █ █ █ █ █ █ █ █ █ █ █ █ █ █ █ █ █ █ █ █ █ █ █ █ █ █ █ █ |
| █ █ █ █ █ █ █ █ █ █ █ █ █ █ █ █ █ █ █ █ █ █ █ █ █ █ █ █ █ █ █ █ █ █ █ |

Thank you for your time!
