# Supplementary material for: Voluntary vs. compulsory student evaluation of clerkships: effect on validity and potential bias
Source: BMC Med Educ. 2018 Jan 5;18:9. doi: 10.1186/s12909-017-1116-8 (PMC5756350; doi:10.1186/s12909-017-1116-8)
Supplement: Supplementary file 2 — Study Design. This file is a representation of the study conduct including information about cohort sizes, response rates, and administration of the bogus item (DOC 47 kb) [file 12909_2017_1116_MOESM2_ESM.doc]

Additional file 2: Study Design

Class of 2016 – N = 49 Students

Seven core clerkships and one vacation block

Class of 2017 – N = 49 Students

Seven core clerkships and one vacation block

Intervention to improve RR: Compulsory approach

Data collected for the whole academic year; 343 invitations sent, 192 responses received, RR* = 56%

Data collected for the first six months of the academic year; 171 invitations sent, 171 responses received, RR* = 100%

Bias question added

Forty-three invitations sent (six students were on vacation), 43 responses received, RR* = 100%

*RR corresponded to AAPOR RR 6 (American Association of Public Opinion Research), where complete and incomplete surveys were counted in and all eligible students were included in the denominator.
