# Supplementary material for: Voluntary vs. compulsory student evaluation of clerkships: effect on validity and potential bias
Source: BMC Med Educ. 2018 Jan 5;18:9. doi: 10.1186/s12909-017-1116-8 (PMC5756350; doi:10.1186/s12909-017-1116-8)
Supplement: Supplementary file 3 — December 2015 Clerkship Evaluation Follow-up Survey. This file presents the follow-up survey that was administered to students in the second cohort who completed the clerkship evaluation form with the bogus item (administered in December 2015). It intended to confirm the bias hypothesis (DOC 26 kb) [file 12909_2017_1116_MOESM3_ESM.doc]

Additional file 3

**December 2015 Clerkship Evaluation**

**Follow-up Survey**

***Dear Class, please answer the two questions below pertaining to your response to item number 8 of the clerkship evaluation. Please circle your answer to these questions or check the box if you do not remember what your response was.***

**The clerkship helped me with my application to the bank:**

***My answer was:***

Strongly disagree: 1

Disagree: 2

Neutral: 3

Agree: 4

Strongly agree: 5

Not Applicable: N/A

I don’t remember what my response was ☐ (skip the below question if you do not remember your answer)

***Please indicate which of the below conditions applies to your answer:***

1. I read fully the statement and answered appropriately because the sentence did not make sense to clerkship evaluation
2. I read fully the statement and answered appropriately because I understood the sentence as being pertaining to clerkship evaluation
3. I did not read fully the statement and I answered randomly
4. I do not remember why I chose this answer
